# Coffee Consumption and the Risk of Depression in a Middle-Aged Cohort: The SUN Project

**DOI:** 10.3390/nu10091333

**Published:** 2018-09-19

**Authors:** Adela M. Navarro, Daria Abasheva, Miguel Á. Martínez-González, Liz Ruiz-Estigarribia, Nerea Martín-Calvo, Almudena Sánchez-Villegas, Estefanía Toledo

**Affiliations:** 1 Department of Preventive Medicine and Public Health, School of Medicine, University of Navarra, 31008 Pamplona, Spain; adela.navarro.e@gmail.com (A.M.N.); dabasheva@alumni.unav.es (D.A.); mamartinez@unav.es (M.Á.M.-G.); lruiz.29@alumni.unav.es (L.R.-E.); nmartincalvo@unav.es (N.M.-C.); 2 Department of Cardiology, Complejo Hospitalario de Navarra, Servicio Navarro de Salud Osasunbidea, 31008 Pamplona, Spain; 3 IdiSNA, Navarra Institute for Health Research, 31008 Pamplona, Spain; 4 Centro de Investigación Biomédica en Red Área de Fisiopatología de la Obesidad y la Nutrición (CIBEROBN), 28029 Madrid, Spain; 5 Department of Nutrition, Harvard TH Chan School of Public Health, Boston, MA 02115, USA; 6 Nutrition Research Group, Research Institute of Biomedical and Health Sciences, University of Las Palmas de Gran Canaria, 35016 Las Palmas de Gran Canaria, Spain; almudena.sanchez@ulpgc.es

**Keywords:** coffee, depression, cohort study

## Abstract

Coffee is one of the most widely consumed drinks around the world, while depression is considered the major contributor to the overall global burden of disease. However, the investigation on coffee consumption and depression is limited and results may be confounded by the overall dietary pattern. We assessed the relationship between coffee intake and the risk of depression, controlling for adherence to the Mediterranean diet. We studied 14,413 university graduates of the ‘Seguimiento Universidad de Navarra’ (SUN) cohort, initially free of depression. We evaluated coffee consumption using a validated food-frequency questionnaire (FFQ). Incident depression cases were adjudicated only if the participant met two criteria simultaneously: (a) validated physician-diagnosed depression together with (b) new onset of habitual antidepressant use. Both criteria were needed; participants meeting only one of them were not classified as cases. Participants who drank at least four cups of coffee per day showed a significantly lower risk of depression than participants who drank less than one cup of coffee per day (HR: 0.37 (95% CI 0.15–0.95)). However, overall, we did not observe an inverse linear dose–response association between coffee consumption and the incidence of depression (*p* for trend = 0.22).

## 1. Introduction

Depression is considered the major contributor to the overall global burden of disease and a common cause of disability worldwide, with more than 300 million people affected [1]. Severe forms of depression can lead to suicide, which is the second leading cause of death in people aged 15–29 years, accounting for 800,000 deaths every year [2]. The lifetime prevalence of depression and the distribution of suicide rates are not uniform. Within Europe, both depression prevalence and suicide rates are higher in northern countries than in southern ones [3]. Nowadays, the prevention of depression represents a public health priority due to its huge social and economic burden.

Some investigations suggest that underlying pathophysiological mechanisms in depression are also present in metabolic syndrome (MetS), obesity, and cardiovascular disease (CVD) [4]. Endothelial dysfunction and an increased production of proinflammatory cytokines may explain the link between depression and CVD [5,6].

On the other hand, coffee is one of the most widely consumed beverages around the world. It is known that coffee contains antioxidant substances with potentially beneficial properties; e.g., chlorogenic acid, flavonoids, melanoidins, and various lipid-soluble compounds such as furans, pyrroles, and maltol [7].

Two recent meta-analyses including three longitudinal and five cross-sectional studies found an inverse association between coffee consumption and depression [8,9]. It is noteworthy that none of the longitudinal studies adjusted their estimates for an overall dietary pattern. Given that coffee consumption may be associated with a high-quality overall dietary pattern and that a healthy dietary pattern, such as the traditional Mediterranean diet (MedDiet), has been associated with a lower risk of depression [10], the overall dietary pattern may be a potential confounder in the association between coffee consumption and depression. Therefore, it is interesting to assess the association between coffee consumption and the risk of depression once adherence to an overall healthy dietary pattern has been accounted for in the analysis. This seems especially relevant when the association is assessed in a Mediterranean setting.

To our knowledge, the effect of coffee on the risk of depression has not been assessed in a Mediterranean cohort and it has neither been assessed if coffee consumption can show an inverse association with depression incidence once adherence to the traditional MedDiet has been accounted for. Thus, the aim of this study was to evaluate whether coffee consumption is independently associated with the risk of depression in the SUN project, a prospective cohort of Spanish graduates, after controlling for adherence to the traditional MedDiet.

## 2. Materials and Methods

### 2.1. Study Population

The “Seguimiento Universidad de Navarra” (SUN) project is a prospective multipurpose cohort of Spanish university graduates. The study methods have been described in more detail elsewhere [11]. Briefly, the SUN project is a dynamic cohort assessing the relationship between diet and chronic diseases. It was developed inspired by the models of the Nurses’ Health Study and the Health Professionals Follow-Up Study. Recruitment started in December 1999 and is permanently open. After the initial questionnaire, follow-up questionnaires are mailed every other year to participants to update information on diet and lifestyle and collect information on health outcomes which might have happened in the previous two years. For participants lost to follow-up, the National Death Index is consulted periodically to assess their vital status. Participants are middle-aged university graduates from different Spanish regions.

By 2017, 22,564 participants were recruited. In order to allow the minimal follow-up of two years, we included only those participants who were recruited before March 2014 (2.75 years before the database closing date). Out of 22,279 eligible subjects, we excluded 1990 participants with no follow-up information (retention rate 91%); 1910 participants with total energy intake out of predefined limits (<500 or >3500 kcal/day for women and <800 or >4000 kcal/day for men); participants with previously diagnosed cardiovascular diseases, cancer, or diabetes (*n* = 1798); participants who died before returning their first follow-up questionnaire (*n* = 39); participants with baseline depression, regular antidepressant use, or implausible date or depression diagnosis (*n* = 1811); as well as patients with diagnosed depression during the first 2 years of follow-up or regular antidepressant use at 2 years of follow-up (*n* = 318). The final sample consisted of 14,413 participants who answered at least 1 follow-up questionnaire.

### 2.2. Assessment of Coffee Consumption

The baseline questionnaire included a previously validated 136-item food-frequency questionnaire (FFQ) [12,13,14]. The serving size for coffee was 50 cc. Information about the consumption of regular and decaffeinated coffee was gathered separately. The FFQ assessed regular food consumption over the previous 12 months and included nine categories of response for the frequency of consumption, ranging from ‘never/seldom’ to ‘more than six times per day’. Then, participants were grouped in four categories according to their level of coffee consumption (<1 cup/day, 1 cup/day, >1–<4 cups/day, ≥4 cups/day).

### 2.3. Case Ascertainment

We adjudicated an incident case of major depressive disorder during follow-up in a participant initially free of any history of depression only if he or she met 2 criteria simultaneously: (a) a validated [15] self-reported new physician-made diagnosis of depression together with (b) new-onset habitual use of antidepressants (in the previous 2 years). Both criteria were needed; participants meeting only one of them were not classified as cases.

### 2.4. Assessment of Covariates

Sociodemographic, anthropometric, lifestyle, and comorbidity information were also collected at baseline and updated every two years through the follow-up questionnaires. The adherence to the MedDiet was established based on the information in the FFQ according to the index defined by Trichopoulou et al. [16]. The latest available information on food composition tables for Spain was utilized by trained dietitians to update the nutrient dataset from the information collected with the FFQ. The baseline questionnaire also included three questions on self-perceived personality traits with scores ranging from 0 to 10. More concretely, these questions assessed self-perceived psychological dependence (0—autonomous to 10—dependent), competitiveness (0—conformist to 10—competitive) and anxiety (0—relaxed to 10—tense) [17].

### 2.5. Statistical Analysis

Baseline quantitative traits of participants were described as the mean and standard deviation according to categories of coffee consumption and baseline qualitative traits, and as the percentage across the same categories. We calculated *p* values for comparisons across categories of coffee consumption with ANOVA for quantitative variables and with chi-squared tests for qualitative variables.

Cox regression models were fit to assess the association between coffee intake and the risk of clinical depression development. We used age as the underlying time variable in all the analyses. Models were stratified by age and period of completion of baseline questionnaire. Participants contributed to the person-years of follow-up from the study inception until diagnosis of depression, death, or last follow-up questionnaire; whichever occurred first.

In our main analysis, we used total coffee intake as the exposure variable. The group in the lowest level of coffee consumption was used as the reference category in all the analyses. For the linear trend test, the median in each category of coffee consumption was calculated to generate a new quantitative variable. As a sensitivity analysis, we also fit models for regular and decaffeinated coffee separately.

The final model was adjusted for potential confounders such as sex, body-mass index (BMI; 3 categories), physical activity (continuous), alcohol intake (linear and quadratic), smoking status (never/former/current/missing) and package-years of smoking (continuous), total energy intake (continuous), adherence to the traditional MedDiet (continuous), years of university studies (continuous), marital status (3 categories), TV-watching hours (continuous), snacking, following any special diet, baseline hypertension and baseline hypercholesterolemia, self-perception of competitiveness, anxiety, and psychological dependence (continuous), and use of tranquilizers or anxiolytic drugs, and was stratified for age (decades) and recruitment period.

The interactions of coffee consumption (4 categories) with sex, age (2 categories), and smoking status (4 categories) were studied by introducing an interaction term in the model and calculating the likelihood ratio test between the model with the interaction and the model without it.

All analyses were performed with Stata SE 15.0. A two-sided *p* value below 0.05 was deemed as statistically significant.

## 3. Results

We followed 14,413 participants, 5765 (40%) men and 8648 women, for a mean follow-up time of 10 years (standard deviation (SD): 4). Mean age of participants at recruitment was 36.4 years (SD: 11.5). Among 144,029 person-years follow-up, we identified 199 incident cases of depression. The incidence rate of depression was 1.3/1000 person-years of follow-up in the lowest category of coffee consumption and 1.5, 1.5, and 0.8/1000 persons-years of follow-up in the subsequent categories.

Participants’ baseline characteristics by category of coffee consumption are shown in Table 1. On average, participants in the highest category of coffee consumption were older, had a higher average BMI, and reported higher mean total energy intake, lower physical activity, and being more tense compared to participants in the lower coffee consumption categories. Those participants were also more likely to be male, married, current smokers, and to consume more alcohol. At baseline, they also reported higher blood cholesterol levels and were more prone to be following any special diet than their peers in the other categories of coffee consumption.

Table 2 presents hazard ratios (HR) and their 95% confidence intervals (CI) for the risk of depression in the crude and multivariable adjusted models. In the comparison across extreme categories of coffee consumption, participants who consumed at least 4 cups of coffee per day showed a 63% (HR = 0.37, 95% CI 0.15–0.95) lower risk of depression than participants who drank less than 1 cup of coffee per day. However, overall, we did not observe a linear dose–response association between coffee consumption and the incidence of depression (*p* for trend = 0.22).

No significant interaction was found between total coffee consumption and sex, age, or smoking status in their association with incident depression (*p* > 0.05 for all of them).

In further analyses, we specifically studied regular and decaffeinated coffee consumption (Table 3). The HR for the risk of depression associated with ≥4 cups per day of regular coffee compared to <1 cup per day was 0.44 (95% CI: 0.18–1.11; *p* for trend = 0.141), in a model adjusted for the consumption of decaffeinated coffee consumption. On the other hand, decaffeinated coffee consumption was not associated with the risk of depression in the fully adjusted model.

## 4. Discussion

In this study, we found that participants who consumed at least four cups of coffee per day showed a lower risk of depression than participants who drank less than one cup of coffee per day. Nevertheless, we found no significant dose–response relationship between coffee consumption and the risk of depression.

The observed inverse association between extreme categories of coffee consumption is consistent with previous literature. On the one hand, several cross-sectional studies have assessed this association [18,19,20,21,22]. Some [18,19,20], but not all [21,22], found a significant inverse association between coffee consumption and the risk of depression. However, due to the cross-sectional design of these studies, reverse causality cannot be ruled out. On the other hand, there are three prospective studies that have longitudinally assessed the association between coffee consumption and the risk of depression [23,24,25]. These three prospective studies have been pooled in two independent meta-analyses [8,9]. The combined results suggested an inverse association between coffee consumption and the risk of depression. As far as the setting for the three prospective cohort studies was concerned, two of them had been conducted in the U.S. [23,24], and another one, including a smaller number (2232) of participants, in Finland [25]. Individually, the three studies described an inverse association between coffee consumption and the risk of depression. The strength of the association was highest in the Finnish cohort, which included only men [25]. In that study, a 75% reduction in the risk of depression was observed when heavy coffee drinkers were compared with non-coffee drinkers [25]. Nevertheless, the analyses were based on 73 events. Risk reductions in the other two cohorts were milder [23,24]. It is worth mentioning that our study had some differential characteristics with previous prospective studies. First, the mean age of participants in our cohort was 36 years, whereas the mean age was 53 years in the study by Ruusunen et al. [25], 62 years in the study with data from the NIH-AARP study [24], and 63 years in the Nurses’ Health Study [23]. Also, in the Finnish study, the outcome was given by a discharge diagnosis of depressive disorder [25] and our outcome—consistent with the other two studies [22,23]—was ascertained through self-reported information.

When we separately assessed the association between regular and decaffeinated coffee consumption and the risk of depression, we found no significant association for decaffeinated coffee consumption. Out of the three longitudinal studies which have assessed the association between coffee consumption and depression risk, decaffeinated coffee consumption was associated with a lower risk of depression (HR ≥ 4 cups/day vs. none = 0.88 (95% CI 0.78–1.00), *p* for trend = 0.003) in the NIH-AARP study [24], but no significant association for decaffeinated coffee consumption was observed in the Nurses’ Health Study [23]. In the Kuopio Ischaemic Heart Disease Risk Factor Study, decaffeinated coffee consumption was not specifically assessed [25].

In a dose–response meta-analysis on coffee consumption and depression risk, Grosso et al. observed a nonlinear J-shaped dose–response association with a peak (the lowest observed risk) for the inverse association at 400 mL/day, which was stable toward a slight increase for higher coffee consumption [9]. It is worth mentioning that the studies that contributed most—i.e., had a higher weight—in this meta-analysis had been conducted in the U.S. [23,24], where the typical serving size is bigger than in Spain. The greatest risk reduction in our study was observed for participants who consumed at least four cups of coffee per day compared to those who consumed less than one cup per day, but we were not able to draw conclusions for participants with heavier coffee consumptions.

There are two main hypotheses which could explain the association between the higher coffee intake and a possible reduction in the risk of depression. First, coffee is the main dietary source of caffeine. Caffeine is an alkaloid exerting a stimulant effect on the central nervous system and modulating the dopaminergic activity by nonspecific antagonism against A1/A2 adenosine receptors. A moderate amount of caffeine has a beneficial effect, improving psychomotor activity, vigilance level, and increasing the perception of feeling more energetic [26]. Second, coffee has a high concentration of polyphenols, such as chlorogenic acid and trigonelline, which have anti-inflammatory potential [7]. Thus, coffee consumption could protect against low-grade inflammation, which seems to be involved in the pathogenesis of depression [27]. In fact, coffee is the main dietary source of polyphenols [28,29] in some populations such as the U.S. or Northern Europe, where the other prospective studies on coffee and depression had been conducted [21,22,23]. Contrarily, in our cohort (data not shown), as well as in other Spanish cohorts, fruits—and not coffee—are the primary source of polyphenols [30]. Interestingly, in the prospective studies conducted so far [23,24,25], the analyses were not adjusted for an overall dietary pattern. Only one analysis [24] was adjusted for daily intake of folate and polyunsaturated fatty acids. Therefore, it was unknown if coffee had the same beneficial effect on participants beyond an overall healthy dietary pattern. To our knowledge, our study is the first one evaluating the association between coffee consumption and depression in which adherence to an overall healthy dietary pattern has been accounted for.

Some limitations of our study should be acknowledged. First, the SUN cohort is not a representative sample of the general population in the pure statistical sense. However, lack of representativeness does not preclude from establishing associations [31,32]. These associations can be generalized to other groups as long as no biological mechanism suggests that the association no longer holds for other populations. Second, dietary information was self-reported. Therefore, we cannot exclude some degree of nondifferential misclassification which could have biased our results more probably towards the null. However, the FFQ has been previously validated [12,13,14]. Third, due to the strict criteria used for the adjudication of the outcome together with some particular characteristics of our study participants—such as their high educational level and their high levels of health-consciousness related to voluntarily participating in a cohort—the incidence of depression in our cohort may seem relatively low in comparison with other studies. However, when we included as incident cases all participants with a medical diagnosis of depression, those who were using antidepressant medication, and the cases that occurred in the two first years, the overall incidence of depression in the cohort during follow-up was 6% (data not shown). In any case, this does not necessarily mean a bias in the sample, as Rothman and other methodologists have repeatedly considered regarding the nonrepresentative nature of most cohorts in the statistical sense of “representativeness” [32]. Fourth, although all the results were adjusted for potential confounders, we cannot exclude the presence of some residual confounding factors that could partly explain our results. Nevertheless, with subsequent adjustment of our models with a wide array of potential confounders, the association became stronger for total coffee consumption and for regular coffee consumption. Therefore, we believe it is unlikely that unmeasured confounders could explained the observed association. Fifth, coffee consumption was assessed only in the baseline questionnaire, assuming it was maintained over time. Nevertheless, previous studies have suggested that coffee consumption remains relatively stable over time [33]. Sixth, tea consumption was not very common in Spain by the time the FFQ was developed, and this item was thus not included in the FFQ. Therefore, we could not assess the specific association between tea consumption and incident depression.

Several strengths of this study deserve to be mentioned. The prospective longitudinal design of the study with an extended follow-up period, the relatively large sample size, the validated assessment of coffee consumption, the validated self-reported medical diagnosis of depression, the ability to control for a good number of potential confounding factors, and the high retention rate (91%) are strengths of our study. Additionally, the high educational level of our participants could contribute to increase the quality of the self-reported information and, thus, reduce the potential for misclassification bias. Furthermore, the exclusion of participants with a depression diagnosis or use of antidepressant medication at baseline or before the first two years of follow-up reduced the possibility of reverse causation bias due to subclinical cases of depression present at baseline. Baseline coffee consumption of participants with baseline depression or antidepressant use might be a consequence of their condition, rather than vice versa. Also, participants who were diagnosed during the first two years of follow-up might have already had some symptoms at the study inception, which might have conditioned their coffee consumption. Therefore, we excluded participants with self-reported depression or antidepressant use during the first two years of follow-up in order to ensure temporal sequence. Finally, the incident cases were defined as self-reported physician-diagnosed depression together with commencement of regular antidepressant medication. Self-reported medical diagnosis of depression showed an acceptable validity in a previous validation study [15]. In the present paper, we increased the specificity of our outcome by including as an additional criterion the commencement of regular antidepressant use. This definition is consistent with previous literature in this area [23] and is stricter. Eventually, this definition might have led to the underestimation of true cases and to a lower sensitivity, but to a higher specificity. Supposedly, with perfect specificity, the nondifferential sensitivity of disease detection would not bias the estimate for the relative risk [34].

## 5. Conclusions

In conclusion, higher coffee consumption was inversely associated with the incidence of depression in a Mediterranean cohort, although the linear dose–response association was not significant. Future studies with longitudinal design and intervention studies would be needed to investigate potential health benefits of coffee consumption.

## Figures and Tables

**Table 1:** Baseline characteristics of participants according to total coffee consumption.

Cups/Day	Total Coffee Consumption				*p* Value
	<1	1	>1 and <4	≥4	
*N*	5253	2667	5928	565	
Age at recruitment	34.5 (11.8)	37.7 (11.9)	37.1 (10.8)	39.5 (11.1)	<0.001
Body-mass index (kg/m^2^)	23.2 (3.4)	23.4 (3.3)	23.5 (3.4)	24.1 (3.7)	0.002
Physical activity in METS	28.7 (26.1)	26.8 (23.9)	25.7 (21.5)	26.4 (24.9)	<0.001
Total energy in kcal/day	2292 (630)	2352 (593)	2406 (598)	2479 (653)	<0.001
Adherence to Mediterranean diet (0–9 score)	4.04 (1.79)	4.36 (1.82)	4.37 (1.78)	4.37 (1.69)	0.157
Alcohol intake in g/day	5.24 (8.32)	7.08 (10.25)	7.28 (10.13)	8.34 (14.2)	<0.001
Years of university education	4.92 (1.48)	5.09 (1.50)	5.13 (1.52)	5.12 (1.58)	0.055
Sex (% male)	41.2	40.1	38.4	45.3	0.001
Snacking (%)	35.8	29.1	32.5	35.4	<0.001
Special diet (%)	6.24	6.60	7.25	9.73	0.007
Hypertension (%)	8.68	9.00	8.11	8.85	0.514
Cholesterol >200 mg/dl (%)	13.0	15.7	16.0	20.2	<0.001
Smoking (%)					
Never	59.5	49.5	41.8	28.8	
Current	19.4	22.2	29.5	40.4	
Former	18.3	25.9	26.4	27.1	<0.001
Marital status (%)					
Single	56.1	42.7	44.2	36.5	
Married	41.6	54.7	53.1	60.7	
Other	2.25	2.55	2.77	2.83	<0.001
Personality traits (range 0–10)					
Psychological dependence	3.69 (2.83)	3.49 (2.87)	3.53 (2.81)	3.59 (2.97)	0.236
Competitiveness	6.99 (1.73)	6.96 (1.77)	6.96 (1.70)	7.09 (1.73)	0.086
Anxiety	5.82 (2.22)	5.80 (2.20)	5.91 (2.13)	6.22 (2.19)	0.017

Data are mean (standard deviation), unless otherwise stated.

**Table 2:** Hazard ratios (HR; 95% confidence intervals) for incidence of depression according to baseline total coffee consumption.

Cups/Day	Total Coffee Consumption				*p* for Trend
	<1	1	>1 and <4	≥4	
Cups/day (median)	0.07	1	2.5	5	
*N*	5253	2667	5928	565	
Cases	64	39	91	5	
Person-years	51,145	26,065	60,705	6115	
Crude HR	1 (ref.)	1.14 (0.76–1.70)	1.12 (0.81–1.55)	0.60 (0.24–1.50)	0.963
Model 1	1 (ref.)	1.12 (0.75–1.67)	1.09 (0.79–1.51)	0.58 (0.23–1.45)	0.923
Model 2	1 (ref.)	1.05 (0.70–1.58)	0.95 (0.68–1.33)	0.37 (0.15–0.95)	0.220

Results from Cox regression models. Age was the underlying time variable in all analyses. Model 1: adjusted for sex and stratified for age (decades) and recruitment period. Model 2: adjusted for sex, alcohol intake (linear and quadratic term), years of university education, marital status, smoking, body mass index, total energy intake, adherence to the Mediterranean diet, between-meal snacking and following special diets, leisure-time physical activity (METS-h/week), hours of TV watching, hypertension at baseline, baseline high blood cholesterol, self-perception of competitiveness, anxiety, and psychological dependence, and use of anxiolytics, and stratified for age (decades) and recruitment period.

**Table 3:** Subgroup analysis. Hazard ratios (95% confidence intervals) for incidence of depression according to baseline regular and decaffeinated coffee consumption.

Cups/Day	Regular Coffee Consumption				*p* for Trend
	<1	1	>1 and <4	≥4	
Cups/day (median)	0	1	2.5	5	
*N*	6315	3433	4193	472	
Cases	84	49	61	5	
Person-years	61,621	34,065	43,130	5212	
Crude HR	1 (ref.)	1.01 (0.71–1.44)	0.97 (0.69–1.35)	0.65 (0.26–1.60)	0.569
Model 1	1 (ref.)	1.00 (0.70–1.42)	0.96 (0.69–1.34)	0.64 (0.26–1.59)	0.533
Model 2	1 (ref.)	0.96 (0.67–1.37)	0.84 (0.59–1.18)	0.43 (0.17–1.07)	0.095
Additionally adjusted for decaffeinated coffee consumption	1 (ref.)	0.97 (0.68–1.39)	0.87 (0.61–1.23)	0.44 (0.18–1.11)	0.141
**Cups/day**	**Decaffeinated Coffee Consumption**				***p* for Trend**
	**<1**		**1**	**>1**	
Cups/day (median)	0		1	2.5	
*N*	12,700		1268	445	
Cases	167		21	11	
Person-years	127,007		12,674	4348	
Crude HR	1 (ref.)		1.25 (0.79–1.96)	1.90 (1.03–3.51)	0.033
Model 1	1 (ref.)		1.20 (0.76–1.89)	1.77 (0.96–3.26)	0.065
Model 2	1 (ref.)		1.20 (0.76–1.89)	1.54 (0.82–2.87)	0.142
Additionally adjusted for regular coffee consumption	1 (ref.)		1.15 (0.72–1.82)	1.46 (0.78–2.76)	0.218

Results from Cox regression models. Age was the underlying time variable in all analyses. Model 1: adjusted for sex and stratified for age (decades) and recruitment period. Model 2: adjusted for age, sex, alcohol intake (linear and quadratic term), years of university education, marital status, smoking, body mass index, total energy intake, adherence to the Mediterranean diet, between-meal snacking and following special diets, leisure-time physical activity (METS-h/week), hours of TV watching, hypertension at baseline, baseline high blood cholesterol, self-perception of competitiveness, anxiety, and psychological dependence, and use of anxiolytics.
